# Motor unit number index (MUNIX) in the D50 disease progression model reflects disease accumulation independently of disease aggressiveness in ALS

**DOI:** 10.1038/s41598-022-19911-0

**Published:** 2022-09-26

**Authors:** Theresa Ebersbach, Annekathrin Roediger, Robert Steinbach, Martin Appelfeller, Anke Tuemmler, Beatrice Stubendorff, Simon Schuster, Meret Herdick, Hubertus Axer, Otto W. Witte, Julian Grosskreutz

**Affiliations:** 1 Department of Neurology, Jena University Hospital, Am Klinikum 1, 07747 Jena, Germany; 2 Precision Neurology, University of Lübeck, Lübeck, Germany; 3 Center for Healthy Ageing, Jena University Hospital, Jena, Germany

**Keywords:** Amyotrophic lateral sclerosis, Neurodegeneration

## Abstract

The neurophysiological technique motor unit number index (MUNIX) is increasingly used in clinical trials to measure loss of motor units. However, the heterogeneous disease course in amyotrophic lateral sclerosis (ALS) obfuscates robust correlations between clinical status and electrophysiological assessments. To address this heterogeneity, MUNIX was applied in the D50 disease progression model by analyzing disease aggressiveness (D50) and accumulation (rD50 phase) in ALS separately. 237 ALS patients, 45 controls and 22 ALS-Mimics received MUNIX of abductor pollicis brevis (APB), abductor digiti minimi (ADM) and tibialis anterior (TA) muscles. MUNIX significantly differed between controls and ALS patients and between ALS-Mimics and controls. Within the ALS cohort, significant differences between Phase I and II revealed in MUNIX, compound muscle action potential (CMAP) and motor unit size index (MUSIX) of APB as well as in MUNIX and CMAP of TA. For the ADM, significant differences occurred later in CMAP and MUNIX between Phase II and III/IV. In contrast, there was no significant association between disease aggressiveness and MUNIX. In application of the D50 disease progression model, MUNIX can demonstrate disease accumulation already in early Phase I and evaluate effects of therapeutic interventions in future therapeutic trials independent of individual disease aggressiveness.

## Introduction

Amyotrophic lateral sclerosis (ALS) is a multisystemic neurodegenerative disease and predominantly characterized by the loss of function of both upper (UMN) and lower motor neurons (LMN). Patients show a high heterogeneity in clinical presentation. Differences in age and site at onset, different patterns of clinical spread and most-of-all variable disease progression-speed hamper care and research. Therefore, reliable surrogate markers are urgently needed to address patient’s individual disease course and enable optimized monitoring for future treatments and trial designs^1,2^.

Motor unit number index (MUNIX) reflects the number of motor units (MU) of LMNs and thereby the loss of function in a measured muscle. It is a non-invasive method and needs only a few minutes of assessment per muscle. For the computation a supramaximal compound muscle action potential (CMAP) and surface EMG interference patterns (SIPs) are recorded during voluntary isometric contractions. Motor unit size index (MUSIX) is the quotient of CMAP (in µV) and MUNIX and indicates reinnervation processes^3–5^. In several recent studies, the technique of MUNIX was validated and established as reliable neurophysiological method in motor neuron diseases including ALS^4–11^. MUNIX is even able to show loss of MU in pre-symptomatic muscles in ALS^12^. Altogether, it has been recognized as a promising biomarker to measure disease progression in ALS^12–15^.

Prior studies used the decline of the revised ALS functional rating scale (ALSFRS-R)^16^ as a parameter for disease progression*,* but had a rather small sample size of the ALS cohort^13–15^. In these studies, MUNIX of distinct muscles respectively summarized as sum scores correlated with the ALSFRS-R score but had a faster decline rate per month than the ALSFRS-R score.

Here we characterized the relation of MUNIX and the quantitative ALS disease course parameters derived from the D50 disease progression model. The model reduces the noise associated with the ALSFRS-R and parameters characterizing the individual disease course can be calculated for any time point^17,18^. It can specifically separate patient’s disease aggressiveness and disease accumulation which is essential to understand the validity of a biomarker in an ongoing clinical trial where patients progress through different phases.

The aim of the study was to clarify whether MUNIX relates to overall disease aggressiveness or to disease accumulation independent of individual progression. We used the D50 disease progression model to analyze separately the relationship between both disease aggressiveness and MUNIX parameters and disease accumulation and MUNIX parameters.

## Materials and methods

### Participants and methods

All participants of this study were recruited from the center for neuromuscular and motor neuron diseases at Jena University Hospital and gave their written and informed consent for participation. The study was approved by the Jena University Hospital Ethics Committee in advance (Nr.3633-11/12) and was performed in accordance with ICH E6 (R2) guideline for good clinical practice. MUNIX measurements of abductor pollicis brevis (APB), abductor digiti minimi (ADM) and tibialis anterior (TA) muscles were conducted between December 2013 and October 2020 by trained and certified clinical neurophysiologists. Previous observations at our department of neurology and prior studies assume that APB, ADM and TA are the most technically feasible and reliable muscles for this method^8–10^. ALSFRS-R were collected in the years 2011 to 2020. In two patients, ALSFRS-R scores have already been collected in 2011 and 2012 before they received the first MUNIX measurement. Those were also included in the calculation of the D50 disease progression model.

The MUNIX method including computation was described in detail previously^3,4,19^. Attention was paid to optimize the position of the CMAP electrode and establish a maximum amplitude. Recording and reference electrodes were self-adhesive (20 × 15 mm, Ambu Neuroline 700). Measurements were performed according to internationally approved protocol and guideline^5,9^.

The healthy controls (n = 45) were mostly spouses of ALS patients, all older than 40 years and did not have any diseases that could influence the measurement. The ALS-Mimics group included patients suffering from spinal canal stenosis with myelopathy (n = 11), paraneoplastic syndrome (n = 7), multifocal motor neuropathy (n = 2), ependymoma (n = 1) and proximal myotonic myopathy (n = 1) with prior differential diagnostic suspicion of ALS and were included during the same time period as ALS patients and controls. 281 ALS patients received MUNIX measurements whilst attending our center for clinical routine. We excluded 44 ALS patients due to one of the following exclusion criteria: Gold Coast diagnostic Criteria for ALS not fulfilled^20^ (n = 36, retrospectively), less than two ALSFRS-R assessments (n = 2), measurements on the clinically more affected side (n = 4) or juvenile ALS (n = 2).

In this study we only considered measurements of the less affected side per muscle and analyzed the first MUNIX measurement of each patient in their individual disease course. Due to the partly already strongly advanced muscle weakness, a value within the inclusion criteria of the MUNIX guideline (CMAP ≥ 0.5 mV)^5^ for all three muscles could not be achieved for each person. That is, some patients had a measurement that met the inclusion criteria of this guideline in only one or two of the three muscles measured. To take that into account and to avoid flawed shifts towards higher median values, we set each measurement without such a value as follows: CMAP = 0.5 mV, MUNIX = 2 and MUSIX = CMAP in µV/MUNIX = 0.5 mV × 1000/2 = 250. We have decided to precisely use these values to avoid technical artifacts on the one hand, but also allow small values on the other. As a result, there were no missing values and no selection bias in the data processed for statistical analyses, because all patients had distinct values for CMAP, MUNIX and MUSIX for each of the three muscles.

### The D50 disease progression model

**Figure 1:**
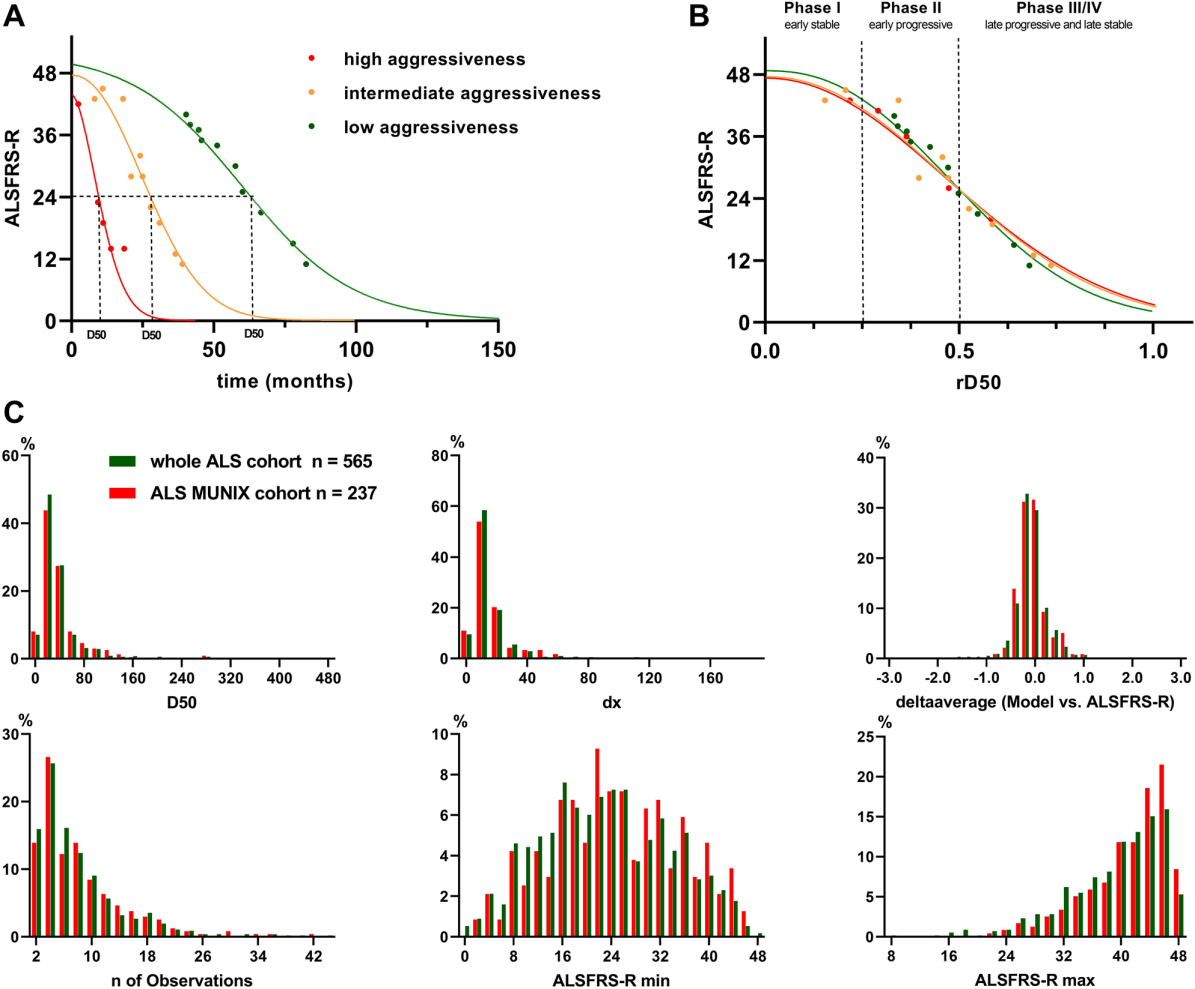
Principles and parameters of the D50 disease progression model: (**A**) calculated sigmoidal curves based on obtained ALSFRS-R scores (dots). D50 represents the individual time cape in months from symptom onset to halved functionality. Three example patients with high (red curve, D50 = 8.92 months), intermediate (yellow curve, D50 = 26.43 months) and low (green curve, D50 = 60.13 months) disease aggressiveness. (**B**) The individual disease duration in reference to D50 yields the parameter relative D50 (rD50). Patients with different D50 values go through similar phases (I–IV) of disease accumulation. rD50 allows to compare patients with vastly different disease aggressiveness. (**C**) Histograms of D50 model parameters of the ALS MUNIX cohort (n = 237, red bars) and of the whole ALS cohort (n = 565, green bars) available at our center.

To address the heterogeneity of the disease in ALS, which complicates comparability among ALS patients and potentially weakens the value of surrogate parameters, nonlinear disease progression in ALS was suggested in previous studies, which utilized generalized additive mixed models, Rasch analysis and longitudinal mixed effect models^21–23^. Here, we applied the D50 disease progression model calculated by an iterative curve fitting approach because it differentiates between disease aggressiveness and disease accumulation^17,24,25^. Based on the ALSFRS-R questionnaires, this model describes the individual disease course as a sigmoidal curve from full health to progressive functional loss. At least two ALSFRS-R questionnaires and the time of the first symptom are necessary for valid modeling. dx represents the decline of the function and highly correlates with D50 (in this cohort R^2^ = 0.94). In detail, D50 is the time taken in months for a patient to lose 50% of his/her functionality (illustrated in Fig. 1A). This parameter (consisting of disease burden over time) is a measure of patient’s disease aggressiveness. Our cohort was divided into three groups based on their D50 values: high (0 ≤ D50 < 20 months), intermediate (20 ≤ D50 < 40) and low (D50 ≥ 40) aggressiveness.

However, disease aggressiveness is only one way of interpreting disease progression. At a certain timepoint (distant from collected ALSFRS-R), patients can be anywhere in their disease trajectory, meaning that different patients show variable neurodegenerative decline at that time. Putting the individual disease duration in reference to D50, our model enables us to replace the time scale with an open-ended scale, where 0 represents disease onset and 0.5 the time point of 50% functionality loss (relative D50, rD50; Fig. 1B). Thereby, patients can be ranged on their individual disease trajectory. Calculation occurred at the time point of MUNIX and represents the individual disease accumulation. Our cohort was divided into the following phases: the early (semi-)stable Phase I (0 ≤ rD50 < 0.25), the early progressive Phase II (0.25 ≤ rD50 < 0.5), and the late progressive and stable Phase III/IV (rD50 ≥ 0.5).

The D50 model provides high precision in Phases I and II during which more than 90% of MUNIX measurements were taken. Later in disease when ALSFRS-R decay drops below 20 and tends to plateau, the model performs with less precision, but only 23 patients of 237 patients had progressed to these later stages of disease^18,24,25^.

Taken together, our model divides the umbrella term “disease progression” into the constructs disease aggressiveness and disease accumulation, whereat the latter provides a standardized measure of disease phases. Also, the D50 model allows quantitative comparisons between different patients in their individual disease course analyzing MUNIX in a pseudo-longitudinal approach.

Figure 1C shows histograms of D50 progression model parameters that illustrate that this ALS MUNIX cohort (n = 237) well represents the regional ALS cohort treated at our center (n = 565). Disease accumulation and disease aggressiveness are independent and separate parameters, which is important to understand patient’s individual disease course in more detail.

### Statistical analysis

All statistical analyses were performed with SPSS Statistics v27.0 (IBM, Chicago, IL, United States). GraphPad Prism v9.0 was used for illustrations (GraphPad Software, San Diego, CA, United States). Normal distribution of data was proofed with the Shapiro–Wilk test. As not all data were normally distributed, but left skewed, comparisons between the three groups were conducted with the Kruskal–Wallis test and post-hoc pairwise comparisons with Bonferroni correction. For differences in nominal variables between groups, the Chi-Square test or Fisher–Freeman–Halton exact test were used. Associations between groups were implemented with a Spearman correlation because data were not following a normal distribution. For all analyses, a p-value less than 0.05 was considered as statistically significant.

## Results

### Participant characteristics

Demographic and MUNIX parameters of the three cohorts are shown in Table 1. The median and percentiles of the controls (Supplementary Table 1) were in accordance with prior studies^26–28^. The controls were slightly younger than the ALS-Mimics (p = 0.047) and the ALS patients (p < 0.001). Gender was also not homogenously distributed over the three groups, there were especially more healthy women in percentage compared to the ALS cohort (p < 0.001).

**Table 1:** Demographics and MUNIX parameters of the three cohorts.

	Controls	ALS	ALS-Mimics
n	45	237	22
**Age**	56.1 (47.7–67.2)	65.8 (58.1–71.9)	62.0 (57.6–75.8)
**Gender f/m**	32/13	103/134	6/16
**APB**			
CMAP	10.4 (8.1–12.2)	4.68 (1.73–7.14)	7.78 (3.54–9.31)
MUNIX	158.7 (120.0–212.1)	51.9 (14.5–106.0)	116.1 (37.6–140.4)
MUSIX	58.7 (53.9–73.1)	87.9 (65.9–183.9)	66.3 (57.5–139.3)
**TA**			
CMAP	5.76 (5.20–6.81)	3.55 (1.03–5.51)	4.46 (0.50–6.41)
MUNIX	132.6 (123.0–149.3)	64.5 (16.4–104.0)	92.1 (2.00–127.2)
MUSIX	42.9 (37.9–49.0)	55.3 (45.7–114.9)	55.1 (46.5–250.0)
**ADM**			
CMAP	10.9 (9.59–12.1)	6.74 (3.18–9.50)	6.95 (1.75–9.04)
MUNIX	147.8 (119.0–186.7)	77.3 (27.2–121.6)	92.3 (3.55–133.3)
MUSIX	69.3 (62.4–80.2)	90.4 (72.1–146.9)	86.5 (70.2–250.0)

Parameters are given as median and interquartile range.

CMAP is given in mV.

*f* female, *m* male.

The whole ALS cohort had a D50 median of 29.2 months (IQR 18.5–46.4 months) and a rD50 median of 0.27 (IQR 0.17–0.39). Detailed clinical data for ALS patients are summarized in Table 2. All measurements with a MUNIX value less than 2 or only a CMAP without MUNIX value were set as small values (CMAP = 0.5 mV, MUNIX = 2, MUSIX = 250). The different ALS phenotypes follow the classification of Chio et al.^29^. The category “Pyramidal ALS” denotes a predominant involvement of the upper motor neurons. All ALS patients fulfilled the Gold Coast criteria of ALS^20^. For better comparison with prior studies, Table 2 also shows the classification according to the revised El Escorial criteria^30^ and the disease progression rate calculated as followed: (48 − ALSFRS-R at MUNIX)/disease duration in months.

**Table 2:** Clinical parameters of the ALS MUNIX cohort.

	Disease accumulation			
	rD50 Phase	I (0 ≤ rD50 < 0.25)	II (0.25 ≤ rD50 < 0.5)	III/IV (rD50 ≥ 0.5)
	n	103	111	23
MUNIX APB		83.9 (32.5–132.2)	44.7 (9.5–80.9)	2.0 (2.0–32.6)
MUNIX TA		93.2 (36.6–132.2)	48.4 (13.1–92.8)	36.7 (2.0–75.3)
MUNIX ADM		93.1 (47.9–137.5)	70.5 (24.1–119.4)	19.7 (8.2–53.1)
MUSIX APB		78.6 (59.5–129.6)	95.1 (71.0–202.5)	250.0 (88.5–250.0)
MUSIX TA		52.0 (44.8–73.9)	55.5 (46.3–152.4)	63.5 (45.9–250.0)
MUSIX ADM		85.2 (67.6–131.8)	92.7 (72.9–161.2)	123.1 (92.3–192.2)
CMAP APB		6.25 (4.19–8.48)	3.79 (1.59–6.42)	0.50 (0.50–3.57)
CMAP TA		4.62 (1.81–6.13)	2.89 (0.82–4.55)	2.32 (0.50–3.35)
CMAP ADM		7.78 (5.45–10.1)	6.33 (3.07–9.20)	2.54 (1.39–6.53)
n of set values	APB/TA/ADM	13/19/13	21/26/17	12/8/4
**D50 disease progression model parameters**				
rD50 at MUNIX		0.17 (0.12–0.21)	0.34 (0.28–0.42)	0.55 (0.53–0.61)
D50 in months		36.8 (21.5–66.1)	26.5 (17.2–40.6)	23.1 (8.6–29.4)
Aggressiveness high/intermediate/low		21/36/46	36/47/28	10/11/2
**Demographic and clinical parameters**				
Age at MUNIX measurement		62.1 (56.0–68.9)	66.7 (59.3–74.9)	66.9 (63.8–74.0)
Gender (female/male)		44/59	48/63	11/12
Disease progression rate*		0.43 (0.20–0.68)	0.65 (0.40–1.13)	1.06 (0.75–2.37)
ALSFRS-R at MUNIX measurement*		43 (41–45)	36 (32–39)	23 (19–25)
n of ALSFRS-R observations		7 (4–12)	5 (3–10)	5 (2–10)
Disease duration at MUNIX		10.4 (7.2–19.3)	17.6 (12.8–28.6)	28.2 (14.2–33.7)
ALS phenotype	Classic	58	66	11
	Bulbar	37	38	11
	Flail arm	3	1	0
	Flail leg	2	0	0
	Pyramidal	3	1	0
	PLMN	0	5	1
Riluzole intake yes/no		92/11	96/15	21/2
Revised El escorial criteria	Definite	28	47	13
	Probable	23	35	3
	LSPR	45	27	7
	Possible	7	2	0

Values are given as median and interquartile range or numbers.

*ADM* abductor digiti minimi, *ALS* amyotrophic lateral sclerosis, *APB* abductor pollicis brevis, *CMAP* compound muscle action potential, *LSPR* laboratory-supported probable, *MUNIX* motor unit number index, *MUSIX* motor unit size index, *PLMN* pure lower motor neuron, *TA* tibialis anterior.

*Related to 213 of the 237 patients in whom the range between MUNIX and ALSFRS-R was 0 ± 4 weeks. ALS phenotypes in accordance to Chio et al.^29^.

For further analysis, the ALS MUNIX cohort was divided into three subgroups based on disease accumulation (rD50) and disease aggressiveness (D50). Stratifying the ALS cohort based on their disease accumulation in three phases of rD50, the D50 medians differed significantly between Phase I and II (p < 0.001). ALS patients with low disease aggressiveness (high D50 value) were in an earlier phase of disease accumulation than patients with a high disease aggressiveness and low D50 value (sample shift^25^).

Gender and phenotype were homogenously distributed throughout the rD50 phases (Chi-Square test, Fisher–Freeman–Halton exact test). However, age was slightly higher for patients in more advanced disease phases (Phase I: 62.08 versus Phase II: 66.67, p = 0.006; Phase I versus Phase III/IV: 66.92 years; p = 0.025).

Age and gender were homogenously distributed throughout the D50 aggressiveness subgroups, only the distribution of phenotypes differed due to more bulbar patients in the high aggressive D50 group in percentage terms (Supplementary Table 2).

### MUNIX parameters reflect disease accumulation

**Figure 2:**
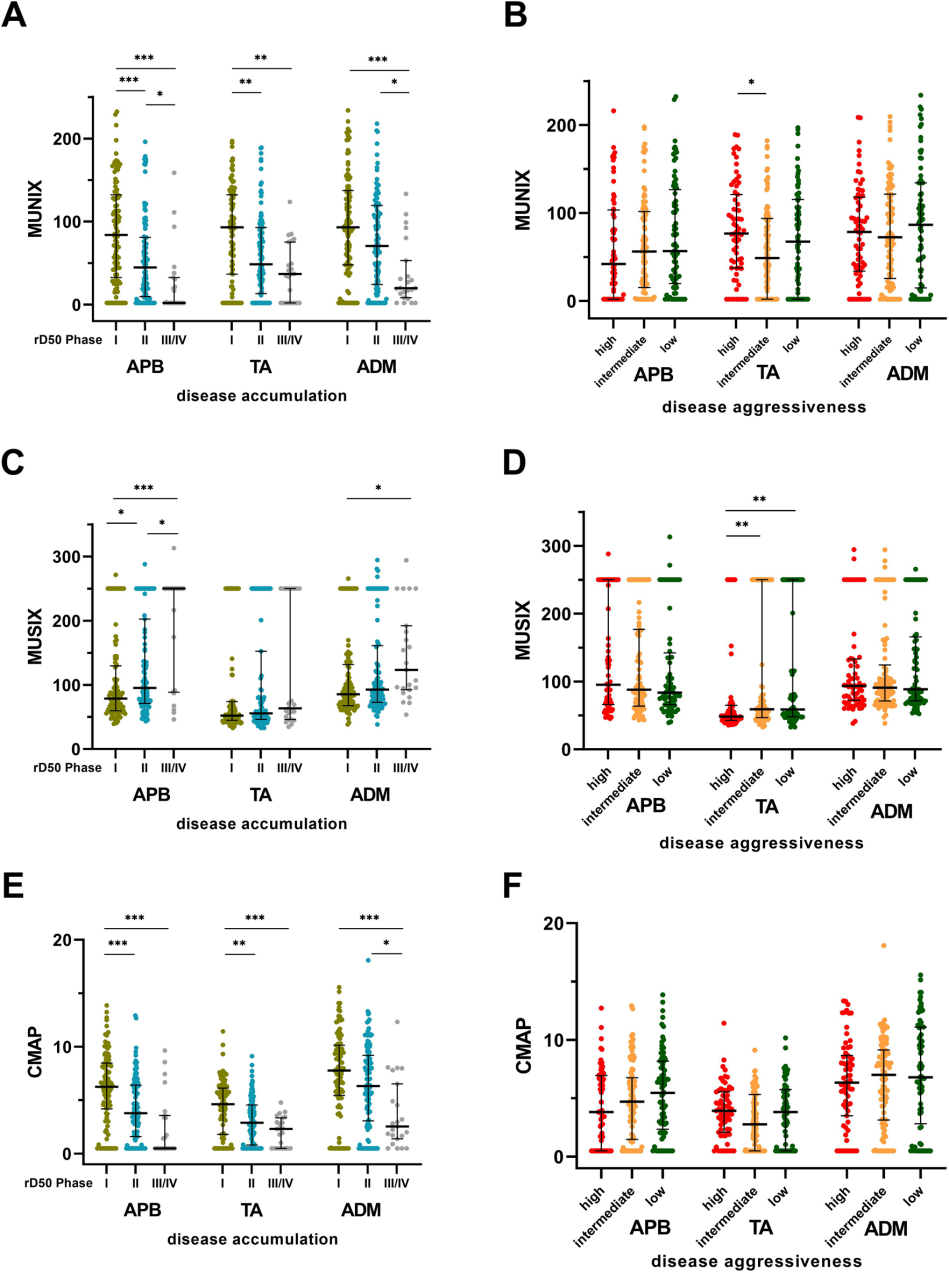
Scatterplots of the ALS cohort of MUNIX (**A,B**), MUSIX (**C,D**) and CMAP (**E,F**) divided for each muscle into three groups based on: (**A,C,E**) rD50 phases: the early semistable Phase I (0 ≤ rD50 < 0.25, in green), the early progressive Phase II (0.25 ≤ rD50 < 0.5, in blue), and the late progressive and stable Phase III/IV (rD50 ≥ 0.5, in gray). (**B,D,F**) High (0 ≤ D50 < 20 months, in red), intermediate (20 ≤ D50 < 40, in yellow) and low (D50 ≥ 40, in green) disease aggressiveness. *p < 0.05, **p < 0.01, ***p < 0.001. Comparisons with Kruskal–Wallis test, pairwise comparisons with Bonferroni correction. Bars indicate median and interquartile range. *ADM* abductor digiti minimi, *APB* abductor pollicis brevis, *CMAP* compound muscle action potential (in mV), *MUNIX* motor unit number index, *MUSIX* motor unit size index, *TA* tibialis anterior.

In statistical analysis MUNIX showed a negative association between different phases for each muscle (APB r = − 0.354, TA r = − 0.294, ADM r = − 0.256; p < 0.001). Stratifying the ALS cohort into three groups based on their phase, significant differences could be found (Fig. 2A).

The MUNIX in APB showed significant differences throughout all disease phases (Phase I versus II and I versus III/IV p < 0.001, II versus III/IV p = 0.019). For MUNIX in TA there were significant differences also between Phase I and II (p = 0.001) and Phase I and III/IV (p = 0.001). There was no significant difference between Phase II and III/IV (p = 0.338). Otherwise, medians of MUNIX in ADM showed no significant differences between Phase I and II (p = 0.068), but between Phase II and III/IV (p = 0.015) and between Phase I and III/IV (p < 0.001).

rD50 subgroup comparisons of CMAP in APB and TA revealed significant differences between Phase I and II (p < 0.001/p = 0.001) and between Phase I and III/IV (p < 0.001), but not between Phase II and III/IV (p = 0.055/p = 0.143, Fig. 2E). CMAP in ADM showed no significant differences between Phase I and II (p = 0.075), but between Phase II and III/IV (p = 0.025) and between Phase I and III/IV (p < 0.001).

For MUSIX in APB there were significant differences between all disease phases (Phase I versus II p = 0.024), I versus III/IV p < 0.001, II versus III/IV p = 0.023, Fig. 2C). The MUSIX in ADM showed only between Phase I and III/IV significant differences (p = 0.014), not between the other phases (Phase I versus II p = 0.386, II versus III/IV p = 0.158). Subgroup comparisons of MUSIX in TA revealed no significant differences.

The percentage of small set values because of “drop-out” below the lower limit increased in each of the three muscles within the rD50 phases. However, this did not differ significantly in TA (p = 0.22) and ADM (p = 0.77) muscles. Whereas in APB, there were significant differences between the percentage of fixed values in Phase I and III/IV (p < 0.001) as well as Phase II and III/IV (p = 0.002).

### MUNIX parameters show independence of disease aggressiveness

Association-analyses between MUNIX of each muscle and D50 did not reveal any significant interaction. Dividing the ALS cohort into three subgroups of disease aggressiveness, there were no significant differences in MUNIX of each muscle except between high and intermediate D50 group of TA (p = 0.036) (Fig. 2B). MUNIX in APB, TA and ADM showed a wide interquartile range for each D50 subgroup.

D50 subgroup comparisons of the CMAP revealed no significant differences in any muscle (Fig. 2F). MUSIX showed no significant differences between all D50 subgroups of APB and ADM, but in TA between both high and intermediate (p = 0.005) and high and low (p = 0.006) D50 subgroups (Fig. 2D).

Medians of MUNIX parameters of each muscle and D50 group are shown in Supplementary Table 2.

### CMAP, MUNIX and MUSIX in ALS, ALS-Mimics and healthy controls

**Figure 3:**
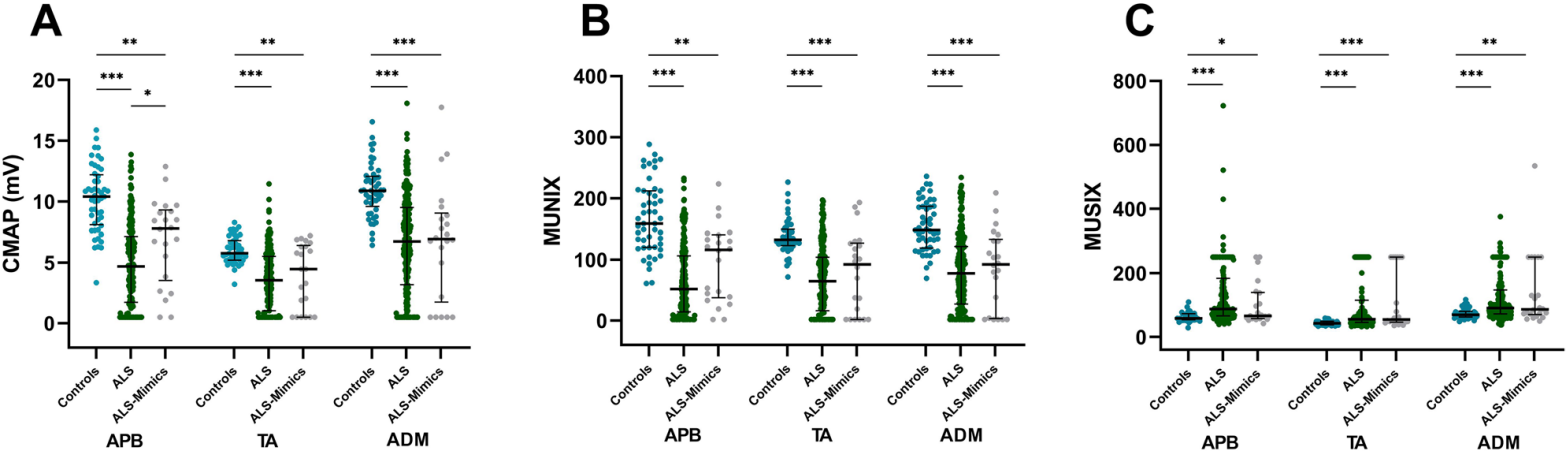
Scatterplots of CMAP (**A**), MUNIX (**B**) and MUSIX (**C**) of APB, TA and ADM of the three different groups. Bars indicate median and interquartile range. *p < 0.05, **p < 0.01, ***p < 0.001. Comparisons with Kruskal–Wallis test, pairwise comparisons with Bonferroni correction. *ADM* abductor digiti minimi, *ALS* amyotrophic lateral sclerosis, *APB* abductor pollicis brevis, *TA* tibialis anterior.

The analysis of CMAP, MUNIX and MUSIX of each muscle resulted in significant differences between controls and ALS patients (p < 0.001) and between controls and ALS-Mimics (p = 0.002) (Fig. 3A–C). ALS and ALS-Mimics showed no significant differences between CMAP, MUNIX and MUSIX values, except for CMAP in APB.

The ALS cohort had the smallest medians of all three CMAP and MUNIX values. The MUSIX median of APB was the highest in the ALS cohort. In TA and ADM muscles the MUSIX median of ALS patients and ALS-Mimics were nearly the same.

## Discussion

In this study we obtained MUNIX parameters in 237 ALS patients. They were analyzed utilizing the D50 disease progression model which provides distinct quantitative measures of patient’s individual disease. These findings show that MUNIX measurements in application of the D50 model can serve as a powerful biomarker indicating individual disease accumulation in patients with ALS independently of individual disease aggressiveness.

In previous MUNIX studies, the decline of the ALSFRS-R was used as a clinical marker for disease progression-speed^12–15^. However, all these studies assume that disease progression in ALS patients follows a linear decline. A few studies suggested a nonlinear disease progression in ALS but did not link this assumption to MUNIX^21–23^. Remarkably, in all these studies, the “disease progression” parameter is seen as one composite marker (of disease aggressiveness and accumulation). In this study, we split this parameter into these two by applying the D50 disease progression model, thus addressing heterogeneity in a sigmoidal approach.

We demonstrate that MUNIX medians show a significant decline above the rD50 phases. In contrast to MUNIX in APB, however, a significant difference for MUNIX medians in ADM occurs firstly between Phases II and III/IV. This mirrors the “split-hand syndrome” of muscle wasting first appearing in the thenar muscle including the APB and affecting the hypothenar muscles like the ADM later in patient’s disease course^31–33^ and is in line with findings in prior studies that also found a smaller decline rate in MUNIX ADM comparing to APB^12,13^. However, the differences in TA between Phase II and III/IV remain non-significant. A reason might be the small group size in this phase. Within the Phases I and II, there is a slight increase in age with a difference in median of around 4 years. As increasing age relates to a decline of CMAP and MUNIX^26,27^, this is a confounder which must be considered, unless the difference of the medians is very small.

MUNIX can therefore be referred to as a marker for the damage of the disease in LMNs. It should be emphasized that the parameter disease accumulation, expressed by rD50 phases, describes a natural number that is independent of disease aggressiveness. This is fundamentally different from previous “staging systems” such as the Milano–Torino Staging System (MiToS)^34^ or King’s staging system^35^, that classify patients into stages from 0 to V, based on the achievement of clinical milestones and the loss of function of key domains of the ALSFRS-R, respectively. The differences of rD50 phases and staging systems were also presented in previous analyses with the D50 disease progression model^24,25^.

CMAP of all muscles behaved very similarly to MUNIX during the phases of disease accumulation. This is not surprising, and this correlation has been shown previously^36^. Since we aimed to address the loss of LMNs in this study, we see the behaviour of CMAP in this context as a supporting component to discuss the conclusions made with MUNIX, but not as a competing or replacing parameter.

MUSIX of APB and ADM showed also nearly the same significant differences between the different phases as MUNIX did and behaved reciprocally to it. Interestingly, there were no significant differences in MUSIX of TA. One reason for this could be the small IQR in Phase I compared to other phases. Although the medians in TA increased slightly, they remained at the same level especially compared to the increase of the MUSIX medians in APB with increasing phases. This suggests, since MUSIX is considered a marker of reinnervation^10^, a lower reinnervation rate during increasing disease accumulation in TA compared with APB and ADM.

Remarkably, the MUNIX and MUSIX medians of APB and ADM show no significant differences throughout the D50 disease aggressiveness subgroups. The significant difference of MUNIX and MUSIX in TA might be an effect of this cohort as there are more bulbar affected patients in the high aggressiveness subgroup in percentage terms. There might be a link between bulbar phenotype and faster disease progression^2^ and therefore higher disease aggressiveness. Taking this into account also the values of TA remain stable through the different disease aggressiveness levels. In summary, MUNIX parameters turn out to be independent of disease aggressiveness. This is also supported by the fact that the CMAP analysis showed no significant differences between D50 subgroups in all muscles. The fact that in TA the differences were also not significant in contrast to MUNIX could be the result of compensatory reinnervation which could smooth the differences. It is known that the CMAP amplitude is affected by these reinnervation processes^12^.

Furthermore, we want to discuss the MUNIX parameters of our ALS cohort in the context of healthy controls and ALS-Mimics. There are significant differences in all three muscles between controls and ALS patients as well as between controls and ALS-Mimics, which is in accordance with prior MUNIX studies^4,15,19^. For the diseases of our ALS-Mimics cohort, few studies with MUNIX exist to date. One study of Philibert et al.^37^ found significant differences in MUNIX APB and ADM in patients with multifocal motor neuropathy compared to healthy controls. Nevertheless, MUNIX parameters are not designed as diagnostic biomarkers because of the widespread of standard deviation of CMAP, MUNIX and MUSIX. In previous studies a wide normal range of these values is also noted^26–28^. Further to this, the differences of ALS and ALS-Mimics are not significant except for CMAP of APB*.* However, CMAP and MUNIX of the ALS group were always the smallest. Reasons for non-significance might be the small sample size of the ALS-Mimics group (n = 22) in comparison to the ALS group (n = 237) and muscle atrophies and loss of MUs in the ALS-Mimics group. On the other hand, the lack of a difference was expected because ALS and ALS-Mimics both suffer from LMN loss. Nevertheless, MUNIX is not formally appropriate to distinguish ALS from ALS-Mimics based on their initial measurement.

As already explained, in some patients, due to the course of the disease, the minimum values according to the guidelines, or MUNIX values at all, could not be achieved. To account for the already advanced loss of LMN function in these cases, values based on the set limits were used. Although the use of MUNIX in CMAP < 0.5 mV is far from ideal, we included the data of low amplitude CMAP to prevent losing ALS patients in advanced disease stage. Regardless of whether these values are excluded or included in the analysis, the key messages remain the same. We consider it essential to include these values, as otherwise falsely high medians will result. The decrease and “drop-out-rate” of MUNIX to a value less than the lower limit of two within the different rD50 phases could be used as a surrogate parameter in future clinical therapy trials. In this ALS cohort, the percentage of “drop-out-rate” increases with higher disease accumulation. An effective therapy could decrease this rate and obtain a higher LMN function over a longer period.

This study is not without limitations. As noted above, controls were 9 years younger compared to ALS patients and ALS patients in Phase I were also slightly younger than those in Phase II. As MUNIX and CMAP decline with aging, the age difference should be kept even smaller in future studies to minimize this potential confounding factor. Furthermore, the group size of ALS-Mimics was small, so these statements should be validated in studies with larger ALS-Mimics cohorts. It was a single center study and single measurements of MUNIX parameters were analyzed. Further analyses with longitudinal data are needed to extent these results. One the other hand, a major advantage of our D50 disease progression model is that longitudinal data, which prolong study duration, is not mandatory as our model enables quantitative comparisons between different patients as a population phenomenon in a pseudo-longitudinal approach. The ALSFRS-R depends on the subjective assessment of the patient, his mood and social environment, on the other hand also on the assessment of the examiner. Nevertheless, we can smooth the noise of the ALSFRS-R with the help of the D50 model.

In summary, MUNIX can detect functional LMN loss in application of the D50 disease progression model in more detail*.* Thereby, MUNIX reflects disease accumulation already in Phase I independently of disease aggressiveness in ALS. The rD50 phase classification, and in particular Phase I, could be used as an inclusion criterion for future studies, as this early phase probably offers the greatest opportunity to intervene therapeutically. MUNIX can therefore be considered an important surrogate parameter to detect MU loss.

## Supplementary Information

Supplementary Tables.

## Data Availability

The datasets generated and analyzed during the current study are available from the corresponding author on reasonable request.

## Abbreviations

ADM: Abductor digiti minimi muscle
ALS: Amyotrophic lateral sclerosis
ALSFRS-R: Revised amyotrophic lateral sclerosis functional rating scale
APB: Abductor pollicis brevis muscle
CMAP: Compound muscle action potential
EMG: Electromyography
IQR: Interquartile range
LMN: Lower motor neuron
LSPR: Laboratory-supported probable
MiToS: Milano–Torino staging system
MU: Motor unit
MUNIX: Motor unit number index
MUSIX: Motor unit size index
PLMN: Pure lower motor neuron
rD50: Relative D50
SIP: Surface EMG interference pattern
SD: Standard deviation
TA: Tibialis anterior muscle
UMN: Upper motor neuron
