# Supplementary material for: Motor unit number index (MUNIX) in the D50 disease progression model reflects disease accumulation independently of disease aggressiveness in ALS
Source: Sci Rep. 2022 Sep 26;12:15997. doi: 10.1038/s41598-022-19911-0 (PMC9512899; doi:10.1038/s41598-022-19911-0)
Supplement: Supplementary file 1 — Supplementary Tables. [file 41598_2022_19911_MOESM1_ESM.pdf]

Motor unit number index (MUNIX) in the D50 disease progression model reflects disease accumulation independently of disease aggressiveness in ALS

Theresa Ebersbach[1,4], Annekathrin Roediger[1,4,*], Robert Steinbach[1], Martin Appelfeller[1], Anke Tuemmler[1], Beatrice Stubendorff[1], Simon Schuster[3], Meret Herdick[3], Hubertus Axer[1], Otto W. Witte[1,2] and Julian Grosskreutz[2,3]

[1] Department of Neurology, Jena University Hospital, Jena, Germany
[2] Center for Healthy Ageing, Jena University Hospital, Jena, Germany
[3] Precision Neurology, University of Lübeck, Lübeck, Germany
[4] These authors contributed equally to the work and share first authorship: Theresa Ebersbach and Annekathrin Roediger

*Correspondence to
Dr. med. Annekathrin Roediger
Department of Neurology, University Hospital Jena
Am Klinikum 1, 07747 Jena
E-Mail: annekathrin.roediger@med.uni-jena.de

Supplementary table 1

Overview of reference values of healthy controls in different studies

**Healthy controls**	**Cao et al. (n = 25*)**	**Delmont et al. (n = 118) First exam**	**Delmont et al. (n = 118) Second exam**	**Jena cohort (n = 45)**		**Neuwirth et al. (n = 66)**	**Delmont et al. (n = 118) First exam**	**Delmont et al. (n = 118) Second exam**
	**Mean ± SD**				**5% - 95% percentiles**			
**APB**								
CMAP	11.0 ± 2.0	9.6 ± 2.9	9.7 ± 2.6	10.2 ± 2.71	6.22 – 14.82	5.1 - 13.4	5.4 - 14.9	6 - 14.7
MUNIX	196.9 ± 48.3	168 ± 68	166 ± 63	168.6 ± 58.6	73.21 – 267.81	78.1 - 247.0	75 - 282	73 - 287
MUSIX	57.9 ± 14.9	61 ± 16	63 ± 17	62.5 ± 13.7	45.67 – 87.55	44.0 - 92.3	43 - 97	42 - 93
**TA**								
CMAP	5.7 ± 0.9	5.6 ± 1.7	5.8 ± 1.7	6.01 ± 1.08	4.49 – 7.85	3.6 - 8.9	3.3 - 9.4	3.3 - 8.7
MUNIX	159.6 ± 27.7	118 ± 36	121 ± 38	137.2 ± 28.9	91.48 – 202.70	69.2 - 195.2	67 - 182	68 - 193
MUSIX	37.1 ± 8.0	48 ± 8	48 ± 9	44.1 ± 6.80	34.47 – 57.41	38.7 - 62.7	38 - 64	38 - 62
**ADM**								
CMAP	9.4 ± 1.7	9.9 ± 2.2	10.1 ± 2.2	10.9 ± 2.17	7.14 – 15.01	7.2 - 13.6	6.9 - 14.4	6.8 - 13.9
MUNIX	166.2 ± 39.1	145 ± 41	150 ± 43	154.4 ± 40.2	90.37 – 223.33	95.3 - 241.8	92 - 223	94 - 238
MUSIX	58.7 ± 12.3	71 ± 15	69 ± 14	73.5 ± 15.7	52.78 – 106.15	47.5 - 97.5	50 - 97	49 - 97
**Age**								
Mean ± SD		74 ± 14	74 ± 14	57.1 ± 9.08	57.1 ± 9.08	49 ± 18.1	74 ± 14	74 ± 14
Range	*50 - 59	23 - 76	23 - 76	40.33 - 71.00	40.33 - 71.00	21–84	23 - 76	23 - 76

[27], [28], [26]. ADM, abductor digiti minimi; APB, abductor pollicis brevis; CMAP, compound muscle action potential; MUNIX, motor unit number index; MUSIX, motor unit size index; SD, standard deviation; TA, tibialis anterior.

Supplementary table 2

Characteristics of the ALS cohort stratified by their D50 subgroups

		Disease aggressiveness High (0 ≤ D50 < 20)	Intermediate (20 ≤ D50 < 40)	Low (D50 ≥ 40)
		**n = 67**	**n = 94**	**n = 76**
D50		13.7 (8.58 - 16.9)	28.3 (23.5 - 32.6)	61.5 (46.7 - 91.0)
MUNIX APB		41.9 (2.00 - 103.5)	56.2 (15.3 - 101.6)	56.7 (19.7 - 126.6)
MUNIX TA		76.7 (37.5 - 121.0)	48.8 (2.00 - 93.8)	67.4 (2.00 - 115.3)
MUNIX ADM		78.5 (33.9 - 118.1)	72.3 (25.7 - 121.5)	86.5 (14.7 - 134.3)
MUSIX APB		95.1 (66.0 - 250.0)	88.1 (63.9 - 176.9)	83.5 (65.9 - 142.3)
MUSIX TA		48.4 (42.8 - 64.8)	59.1 (47.0 - 250.0)	58.6 (48.0 - 250.0)
MUSIX ADM		93.6 (72.3 - 133.1)	90.8 (71.4 - 124.4)	88.6 (71.7 - 165.7)
CMAP APB		3.82 (0.5 - 6.95)	4.71 (1.48 - 6.77)	5.47 (2.35 - 8.18)
CMAP TA		3.93 (2.08 - 5.55)	2.78 (0.5 - 5.32)	3.82 (0.5 - 5.75)
CMAP ADM		6.34 (3.50 - 8.67)	7.00 (3.14 - 9.14)	6.79 (2.83 - 11.10)
n of set values	APB/TA/ADM	18/9/10	17/24/11	11/20/13
rD50 at MUNIX		0.34 (0.23 - 0.45)	0.28 (0.18 - 0.40)	0.21 (0.12 - 0.33)
n of rD50 phase (I/II/III-IV)		21/36/10	36/47/11	46/28/2
ALS phenotype	Classic	33	54	48
	Bulbar	32	35	19
	Flail Arm	0	1	3
	Flail Leg	0	0	2
	Pyramidal	0	1	3
	PLMN	2	3	1

Values given as medians and interquartile range. D50 is given in months. ADM, abductor digiti minimi; APB, abductor pollicis brevis; CMAP, compound muscle action potential; MUNIX, motor unit number index; MUSIX, motor unit size index; PLMN, pure lower motor neuron; TA, tibialis anterior. ALS phenotypes in accordance to Chio et al.[29].
